# Review of electrophysiological models to study membrane potential changes in breast cancer cell transformation and tumor progression

**DOI:** 10.3389/fphys.2025.1536165

**Published:** 2025-03-05

**Authors:** Chitaranjan Mahapatra, Arnaw Kishore, Jineetkumar Gawad, Ahmed Al-Emam, Riad Azzam Kouzeiha, Maher Ali Rusho

**Affiliations:** ^1^ Paris Saclay Institute of Neuroscience, Paris Saclay University, Saclay, France; ^2^ Microbiology and Immunology, Xavier University School of Medicine, Aruba, Netherlands; ^3^ Department of Pharmaceutical Chemistry, VIVA Institute of Pharmacy, Virar, India; ^4^ Department of Pathology, College of Medicine, King Khalid University, Asir, Saudi Arabia; ^5^ Faculty of Medical Sciences, Lebanese University, Hadath Campus, Beirut, Lebanon; ^6^ Department of Biomedical Engineering, University of Colorado Boulder, Boulder, CO, United States

**Keywords:** breast cancer, membrane potential, ion channels, gap junction, calcium dynamics, electrophysiological model

## Abstract

The transformation of normal breast cells into cancerous cells is a complex process influenced by both genetic and microenvironmental factors. Recent studies highlight the significant role of membrane potential (Vm) alterations in this transformation. Cancer cells typically exhibit a depolarized resting membrane potential (RMP) compared to normal cells, which correlates with increased cellular activity and more aggressive cancer behavior. These RMP and Vm changes are associated with altered ion channel activity, altered calcium dynamics, mitochondrial dysfunction, modified gap junction communication, and disrupted signaling pathways. Such fluctuations in RMP and Vm influence key processes in cancer progression, including cell proliferation, migration, and invasion. Notably, more aggressive subtypes of breast cancer cells display more frequent and pronounced Vm fluctuations. Understanding the electrical properties of cancer cells provides new insights into their behavior and offers potential therapeutic targets, such as ion channels and Vm regulation. This review synthesizes current research on how various factors modulate membrane potential and proposes an electrophysiological model of breast cancer cells based on experimental and clinical data from the literature. These findings may pave the way for novel pharmacological targets for clinicians, researchers, and pharmacologists in treating breast cancer.

## 1 Introduction

Cancer remains a major global health issue, causing millions of deaths each year and placing a substantial social and economic strain on communities worldwide. This deadly disease is characterized by the uncontrolled growth of abnormal cells that can invade nearby tissues and spread to other body parts, known as metastasis. The global burden of cancer is immense, with an estimated 19.3 million new cases diagnosed in 2020 alone (Sung et al., 2021). Among the various types of cancer, breast cancer (BC) is the most common, accounting for approximately 2.3 million new cases in 2020, making up 11.7% of all cancer cases globally (Sung et al., 2021). BC disproportionately affects women, although men can also be diagnosed with the disease. The global health impact of BC is severe, as it leads to more than 685,000 deaths annually (Mubarik et al., 2023). Breast cancer also remains a major global health issue, significantly affecting both economic systems and public health, as reflected by disability-adjusted life years (DALYs). Over the 3 decades from 1990 to 2021, the number of breast cancer cases worldwide surged from approximately 870,000 to over two million (Li et. al. 2025). Likewise, mortality rates escalated from 350,577 to 660,925 deaths within the same timeframe. By 2021, breast cancer was responsible for an estimated 20.25 million DALYs globally, with an age-standardized DALY rate of 455.56 per 100,000 people (Li et. al. 2025). BC is categorized into several subtypes, including hormone receptor-positive, HER2-positive, and triple-negative BC, each requiring specific treatment strategies. Ductal carcinoma, the most common type of BC, originates in the epithelial cells lining the milk ducts of the breast (Guinebretiere et al., 2005). This form of carcinoma can be categorized into ductal carcinoma *in situ* (DCIS), a non-invasive form where the cancer cells remain confined within the ducts, and invasive ductal carcinoma (IDC), where the cancer cells breach the ductal walls and invade surrounding breast tissue (Udayasiri et al., 2023). The relationship between breast carcinoma and cell physiology is deeply intertwined, as alterations in the normal cellular processes, such as cell proliferation, apoptosis, and differentiation, play a critical role in the development and progression of this cancer (Feng et al., 2018). Membrane potential is the electrical gradient across a cell’s membrane, generated by the differential distribution of ions such as sodium, potassium, calcium, and chloride (McCormick, 2014). In excitatory cells, like neurons, this potential is actively maintained and changes to transmit signals, while in non-excitatory cells, it helps regulate cell proliferation and apoptosis. In cancer, alterations in membrane potential can disrupt normal cellular functions, promoting uncontrolled cell growth and metastasis (Sundelacruz et al., 2009). Changes in membrane potential particularly membrane depolarization, ion channel activity, and signal transduction pathways can disrupt normal cell function, leading to uncontrolled cell growth and the potential for metastasis (Kunzelmann, 2005). Understanding these physiological changes is crucial for developing targeted therapies that can correct or inhibit the abnormal cellular behaviors driving breast carcinoma. Calcium dynamics play a pivotal role in this membrane potential regulation, with the endoplasmic/sarcoplasmic reticulum (ER/SR) serving as a key reservoir for calcium ions (Mahapatra et al., 2024). The release and uptake of calcium from the ER modulate intracellular calcium levels, influencing membrane potential and signaling pathways that control cell proliferation and apoptosis (Pinton et al., 2008). Mitochondria also contribute to this balance by buffering intracellular calcium and generating ATP, which powers ion pumps and channels (Garbincius and Elrod, 2022). Disruptions in these mechanisms, such as altered ion channel expression or dysfunctional calcium handling by the ER and mitochondria, can lead to abnormal membrane potentials, promoting the transformation of normal breast cells into cancerous ones (Bortolin et al., 2022). Gap junctions, composed of connexin proteins, enable direct communication between breast cells by allowing the passage of ions and small molecules, thereby helping to synchronize membrane potentials across cells (Mateos et al., 2024). Disruption of gap junctional communication can lead to altered membrane potential regulation, contributing to uncontrolled cell proliferation and cancer progression in breast cells (Trosko and Chang, 2001). Despite advances in treatment, there remains an urgent need for fundamental and translational research to better understand the underlying mechanisms of BC, improve early detection, and develop more effective therapies to reduce the global health and economic burden of this disease. Investigating membrane electrical activities has emerged as a promising area of research, offering new insights into the role of ion channels and membrane potential in cancer and other pathophysiological conditions. Targeting these electrical properties pharmacologically could lead to novel treatments that specifically disrupt cancer cell proliferation and metastasis while sparing normal cells (Prevarskaya et al., 2018; Li and Xiong, 2011). Scientific review articles are crucial for synthesizing existing research, and providing researchers and clinicians with a comprehensive understanding of complex pathophysiological conditions. These reviews distill key findings, identify knowledge gaps, and highlight potential treatment strategies, ultimately guiding more informed and effective clinical decision-making. Unfortunately, there is currently no comprehensive review that specifically addresses the relationship between membrane potential and cancer in breast cells. This lack of updated information leaves a significant gap in our understanding of how cellular electrophysiology influences cancer progression in these cells. Our review aims to fill this critical gap by synthesizing recent research on membrane potential, ion channel dynamics, and their roles in breast cell transformation and cancer development. This study offers essential insights that will inform future research and guide BC therapeutic approaches, benefiting foundational researchers and clinicians.

## 2 Materials and methods

We extensively searched the MEDLINE database via PubMed, concentrating on English-language publications across all periods (Motschall and Falck-Ytter, 2005). Our objective was to investigate the connections between different types of breast cancer cells and factors such as ion channels, membrane biophysics, gap junctions, mitochondria, calcium dynamics, and intracellular electrical activities (including depolarization, repolarization, hyperpolarization, and resting membrane potential). This review encompassed both experimental and computational studies. To ensure relevance and accuracy, we excluded non-English articles and those that duplicated information. Preference was given to the most recent and detailed manuscripts in cases of overlap. The inclusion criteria covered original research articles, clinical trials (randomized and non-randomized), experimental investigations, observational studies (prospective and retrospective), case-control studies, and reviews examining the influence of various factors on ion channels and membrane potential. Each selected research underwent thorough evaluation, and additional references were incorporated for a comprehensive understanding. Finally, we developed a conceptual model to represent the critical interactions between cellular and subcellular components and their influence on membrane potential, a key process in transforming normal breast cells into cancerous ones.

## 3 Membrane potential

The membrane potential refers to the electrical difference across a cell’s plasma membrane, resulting from unequal ion distributions inside and outside the cell. Measured in millivolts (mV), this voltage difference is typically negative at rest, as intracellular negative charges exceed those outside. This gradient is crucial for action potential generation, signal transmission, nutrient transport, cell volume regulation, and proliferation. Ion movement maintains the membrane potential, which is essential for both excitable cells, like neurons and muscle cells, and non-excitable ones, such as epithelial cells. RMP is the membrane potential value at rest or in passive condition (Sundelacruz et al., 2009). Normal and cancerous cells differ significantly in their membrane potentials/RMPs. While healthy cells generally remain hyperpolarized (more negative to RMP), ensuring controlled growth and intercellular communication, cancer cells often exhibit depolarization (less negative potential) (Shrivastava et al., 2024). Table 1 illustrates the value of RMPs in various excitable and non-excitable cells. This depolarization reflects disrupted ion transport and cellular homeostasis, which promotes unregulated proliferation, resistance to apoptosis, and increased migratory behavior—key traits of malignancy.

**TABLE 1:** Illustrates the RMP values in different excitable, non-excitable, and cancer cells.

Tissue type	RMP (mV)
Smooth Muscles	−80 to −40 mV (Ozaki et al., 1991; Koh et al., 2012)
Cardiac Muscles	−90 to −50 mV (Legato and Bilezikian, 2004; Grant, 2009)
Neuronal Cells	−70 mV (Khadria, 2022)
Pancreatic Beta cells	−70 to – 60 mV (Jacobson and Philison, 2007)
Ovarian Tumor cells	−5 mV (Yang and Brackenbury, 2013)
Leukemic myeloblast	−5 mV (Yang and Brackenbury, 2013)
Human hepatoma	−15 mV (Yang and Brackenbury, 2013)
Cervix Tumor	−15 mV (Yang and Brackenbury, 2013)
PC-3M Prostate Cancer	−55 mV (Yang and Brackenbury, 2013)

**FIGURE 1:**
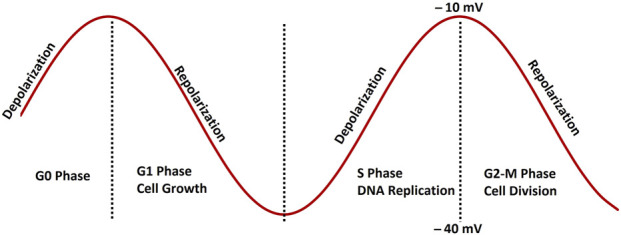
A schematic representation of membrane potential fluctuations during the cell cycle, governed by the synchronized activity of ion channels. This regulation plays a vital role in enabling the progression from the G0/G1 phase to the S phase. During the S phase, the membrane potential generally moves toward depolarization. As the cell enters mitosis, further depolarization continues until cell division is finalized. Following this, the membrane potential reverts to a repolarized state typical of the G0/G1 phase. Detailed explanations are provided in the previous section.

Membrane potential depolarization has been linked to cancer progression across various tissue types, including breast, liver, and ovarian cells. Vm depolarization not only drives tumor progression but also triggers DNA synthesis and mitosis (Sheth and Esfandiari, 2022). The eukaryotic cell cycle consists of G1, S, G2, and M phases, with mitosis (M phase) typically followed by cytokinesis and DNA replication occurring during the S phase (Wang, 2022). At the G1/S transition, Vm depolarization prepares cells to enter the M phase, and, quiescent cells in the G0 phase display increased mitotic activity following Vm depolarization, reinforcing its role in regulating cell division (Patil and Kunda, 2022). In metastasis, tumor cells lose adhesion, migrate, invade, and travel through vascular or lymphatic systems to form secondary tumors. Vm plays a critical role in cell migration by regulating ion and water movement, crucial for metastatic progression (Majidpoor and Mortezaee, 2021). Figure 1 provides a schematic depiction of how membrane potential fluctuates during the cell cycle. These variations are regulated by the synchronized opening and closing of specific ion channels. This coordination plays a vital role in enabling the progression from the G0/G1 phase to the S phase. During the S phase, the membrane potential usually becomes more depolarized. Similarly, mitosis is marked by additional depolarization, which continues until cell division concludes (Cone and Clarence, 1970). Following this, the membrane potential reverts to a repolarized state typical of the G0/G1 phase. In BC cells, depolarization of the membrane potential is often associated with an increased rate of cell proliferation (Strobl et al., 1995). This is partly due to the influence of membrane potential on the activity of cyclin-dependent kinases (CDKs) and other cell cycle regulators (Blackiston et al., 2009). Depolarized membrane potentials can also enhance the uptake of nutrients and growth factors, further promoting the proliferation of cancer cells. For instance, the activity of sodium channels, which contribute to membrane depolarization, has been linked to increased cancer cell growth by activating various signaling pathways, including the MAPK and PI3K/Akt pathways (Iorio et al., 2019). Apoptosis, or programmed cell death, is a crucial mechanism for eliminating damaged or abnormal cells. In cancer, the evasion of apoptosis is a hallmark of disease progression. Membrane potential plays a critical role in regulating apoptosis, with alterations often leading to resistance to cell death in BC cells (Bortner and Cidlowaski, 2014). Hyperpolarization and repolarization of the membrane potential are generally associated with pro-apoptotic signals, whereas depolarization can contribute to anti-apoptotic mechanisms (Wang, 2004).

In BC, the dysregulation of ion channels that control membrane potential can lead to the inhibition of apoptosis, allowing cancer cells to survive and proliferate despite therapeutic interventions (Tajbakhsh et al., 2018). Metastasis, the spread of cancer cells from the primary tumor to distant organs, is a leading cause of cancer-related mortality. Membrane potential alterations are closely linked to the metastatic potential of BC cells (Yang and Brackenbury, 2013). Depolarized membrane potentials are often associated with enhanced cell motility, invasiveness, and the ability to traverse the extracellular matrix. The RMP in breast cancer cells varies depending on the cell type and aggressiveness. For triple-negative breast cancer cells (MDA-MB-231, MDA-MB-468, and MCF-7), the RMP ranges between −40 mV and −20 mV, reflecting their unique electrical properties compared to normal cells (Quicke et al., 2022; Yang and Brackenbury, 2013). In breast cancer cells isolated from patients, the RMP is reported to be approximately −13 mV, which is significantly depolarized compared to normal mammary epithelial cells (Berzingi et al., 2016).

## 4 Ion channels and breast cancer

Ion channels and transporters are fundamental in regulating membrane potential by controlling ion movement across the cell membrane. Key players in this process include potassium (K^+^) channels, sodium (Na^+^) channels, and calcium (Ca^2+^) channels. K^+^ channels allow the outward flow of K^+^ ions, which helps maintain a negative RMP. Na^+^/K^+^ pumps actively move Na^+^ out and K^+^ into the cell, utilizing ATP to sustain the ion gradients necessary for a stable membrane potential (Morth et al., 2011). Other transporters, such as Ca^2+^ pumps and chloride (Cl^−^) channels, contribute to this balance by influencing ion distribution and signaling within the cell (Stauber et al., 2012). These systems collectively ensure the maintenance of RMP, crucial for normal cellular activities and environmental responsiveness. However, in cancer cells, ion channels often display abnormal expression, such as overexpression or dysregulation. This can lead to depolarization, commonly caused by the overactivity of Na^+^, Ca^2+^, or Cl^−^ channels, disrupting normal ion homeostasis (Lastraioli et al., 2015). Conversely, hyperpolarization may occur due to excessive K^+^ efflux through overactive K^+^ channels, resulting in a more negative intracellular state. These alterations in membrane potential significantly impact cellular signaling pathways, supporting cancer cell survival and proliferation.

Potassium channels are vital for maintaining cellular homeostasis and play critical roles in both excitable and non-excitable cells. Among these, voltage-gated K^+^ (Kv) channels have emerged as key contributors to BC progression. Aberrant expression of Kv10.1 (Ether-à-go-go-1, KCNH1, Eag1) is associated with increased tumor aggressiveness and poor clinical outcomes (Ouadid-Ahidouch et al., 2016). Kv10.1 channels, characterized by six transmembrane segments, a voltage-sensing domain, and a pore-forming domain, influence RMP and promote Ca^2+^ influx in cancer cells, supporting cell division and migration (Sahoo et al., 2022). Kv10.1 also aids cell cycle progression by stabilizing the membrane potential in the G1 phase and enhances invasiveness by modulating focal adhesions (Hartung, 2011). Additionally, its expression contributes to cancer cells’ resistance to apoptosis, with over 70% of BC tissues showing abnormal Kv10.1 expression (Lastraioli et al., 2015). Calcium-activated potassium (KCa) channels also play a significant role in cancer by regulating proliferation, migration, and oncogenesis. They are classified into three subtypes based on conductance: large (BK), intermediate (IK), and small (SK) (Mahapatra et al., 2018). BK channels (KCNMA1) are linked to estrogen receptor expression and brain-metastasizing BC cases, while IK/SK channels (KCNN4) correlate with high-grade, lymph node-negative tumors (Brevet et al., 2008; Haren et al., 2010). Other K^+^ channels, such as inward rectifier channel Kir3.1 (KCNJ3) and two pore domain channel K2P9.1 (KCNK9), are associated with lymph node metastasis and proto-oncogenic activity, respectively, with K2P9.1 showing gene amplification in 10% of BC cases (Stringer et al., 2001; Mu et al., 2003). Voltage-gated Na^+^ channels (Nav) were among the earliest ion channels identified as abnormally expressed in BC (Roger et al., 2003). The primary variant implicated in BC is the “neonatal” splice form of SCN5A, known as nNaV1.5 (Fraser et al., 2005). Research indicates that Nav1.5 activity facilitates metastasis, with nNaV1.5 being significantly upregulated in metastatic BC tissues (Brackenbury et al., 2007). Calcium channel dysregulation also characterizes BC, with variations in calcium signaling observed across different BC subtypes. Notably, T-type voltage-gated Ca^2+^ channels (CaT) play a critical role in regulating BC cell proliferation. Interestingly, the mRNA expression of the Cav subunit encoded by CACNA2D3 (α2δ3 subunit) is generally elevated in BC but shows reduced levels in some metastatic cases (Palmieri et al., 2012). Additionally, the secretory pathway Ca^2+^ ATPase I (SPCA1, ATP2C1) is highly expressed in basal-like BCs, and silencing SPCA1 in the basal-like BC cell line MDA-MB-231 leads to reduced proliferation. Conversely, overexpression of the Ca^2+^ efflux pump PMCA2 (ATP2B2) is more strongly linked to HER2 receptor-positive BCs (Grice et al., 2010; VanHouten et al., 2010).

Several transient receptor potential (TRP) channels are significantly overexpressed in BC, with distinct roles in its progression. For instance, TRPM7 shows elevated immunohistochemical expression, particularly in highly proliferative and high-grade BC. This overexpression is linked to metastasis, as high TRPM7 mRNA levels are associated with distant metastases and poor survival outcomes (Guilbert et al., 2009; Middelbeek et al., 2012). TRPV6, another TRP channel, is overexpressed in progesterone receptor and estrogen receptor-negative BC (Bolanz et al., 2008). Studies confirm elevated TRPV6 levels in certain ductal BC biopsies, often correlating with basal-like subtypes, ER-negativity, and worse prognosis (Dhennin-Duthille et al., 2011). Conversely, TRPC1, found in BCs with lower proliferation rates, may not be a suitable target for treating aggressive forms (Dhennin-Duthille et al., 2011). TRPM8 is predominantly expressed in ER-positive, well-differentiated, and lower-grade BCs (Chodon et al., 2010). TRPC6 mRNA levels are notably elevated (up to 200-fold) in BC tissues compared to controls, but its clinical significance remains unclear (Dhennin-Duthille et al., 2011; Aydar et al., 2009). Additionally, ORAI1 and STIM1, components of the store-operated channels (SOC3) family, are upregulated in basal-like BC, which often has a poor prognosis (McAndrew et al., 2011). Basal-like BCs also exhibit lower STIM2 levels, and an STIM1-high/STIM2-low profile indicates increased aggressiveness (Motiani et al., 2013). The proton channel Hv1 (HVCN1) is overexpressed in metastatic BC, with higher levels linked to disease progression and poor outcomes (Wang et al., 2012). The Ca^2+^-activated Cl^−^ channel ANO1 facilitates the progression of breast cancer by triggering the activation of EGFR (epidermal growth factor receptor) and CAMK (calcium/calmodulin-dependent protein kinase) signaling pathways (Qu et al., 2014). This process contributes to cancer cell proliferation and tumor advancement, with studies highlighting its overexpression and amplification in breast cancer tissues and its correlation with disease severity. Piezo1, a mechanosensitive ion channel, plays a significant role in breast cancer progression by enabling cells to detect and respond to mechanical cues. Research has demonstrated that Piezo1 forms functional ion channels in MCF-7 breast cancer cells, and its elevated expression is associated with reduced overall survival in breast cancer patients (Li et al., 2015). Mechanosensitive ion channels like Piezo1 allow cancer cells to sense and adapt to the mechanical properties of their environment, such as stiffness and extracellular matrix composition. This mechanosensation facilitates processes like cell migration, invasion, and metastasis, contributing to cancer progression. By detecting nanomechanical cues through channels like Piezo1, breast cancer cells can modulate their behavior to favor proliferation and metastatic spread (Magazzù and Marcuello et al., 2023). Understanding the role of mechanosensitive ion channels in cancer biology may offer new avenues for therapeutic intervention. The Piezo1 ion channel is encoded by the PIEZO1 gene. Activation of Piezo1 typically leads to membrane depolarization by allowing cation influx, primarily calcium (Ca^2+^) and sodium (Na^+^), into the cell (Gottlieb and Frederick, 2012). Various ion channels involved in regulating membrane potential are abnormally expressed or overexpressed in BC listed in Table 2. Depolarization, repolarization/hyperpolarization are linked to the inward flow of that particular ion via the ion channel.

**TABLE 2:** List of ion channels expressed or overexpressed in breast cancer cells.

Ion channel	Ion channel gene	Effect on membrane potential
Potassium	Kv10.1(KCNH1)	Repolarization/hyperpolarization
Potassium	BK (KCNMA1)	Repolarization/hyperpolarization
Potassium	SK/IK (KCNN4)	Repolarization/hyperpolarization
Potassium	Kir3.1 (KCNJ3)	Repolarization/hyperpolarization
Potassium	K 2P 9.1 (KCNK9)	Repolarization/hyperpolarization
Sodium	Na_V_1.5 (SCN5A)	Depolarization
Calcium	CaT (CACNA2D3)	Depolarization
TRP	TRPM7	Depolarization
TRP	TRPV6	Depolarization
TRP	TRPM8	Depolarization
TRP	TRPC6	Depolarization
SOC	ORAI1	Depolarization
SOC	STIM1	Depolarization
Proton	Hv1 (HVCN1)	Depolarization
Chloride	ANO1	Repolarization/hyperpolarization
Piezo1	PIEZO1	Depolarization

## 5 Gap junction and breast cancer

**FIGURE 2:**
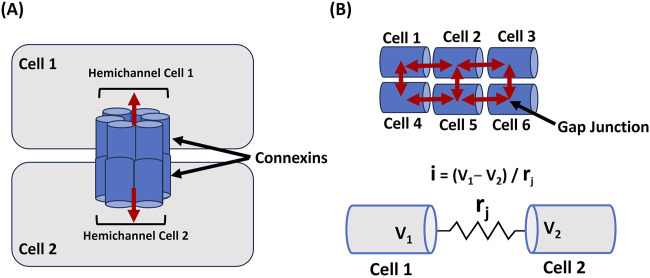
Cellular Coupling via Gap Junctions **(A)** illustrates the formation of a gap junction between Cell 1 and Cell 2 through connexins, facilitating signal exchange, as shown by the red bidirectional arrow. In **(B)**, 6 cells are arranged linearly and interconnected by gap junctions (red arrow), demonstrating signal propagation across the network. The lower section presents an electrical schematic of the gap junction linking Cell 1 and Cell 2, where V1 and V2 represent their respective membrane potentials, and rj denotes the resistance within the gap junction. Detailed explanations are provided in the previous section.

Gap junctions are essential for maintaining the electrical functionality of excitable cells, including neurons and cardiac and smooth muscle cells, by enabling direct intercellular communication. These specialized membrane channels allow ions and small molecules to flow between neighboring cells, supporting synchronized electrical activity (Meşe et al., 2007; Söhl et al., 2005; Kumar and Norton, 1996). Through the rapid, coordinated spread of membrane depolarization and action potentials, gap junctions facilitate the efficient transmission of electrical signals within cell networks. These junctions’ permeability and regulatory properties significantly affect the electrical dynamics of excitable cells. Connexins (Cx) are proteins that assemble to form hemichannels, which link adjacent cells when paired across cell membranes. These paired hemichannels create gap junctions, enabling direct communication and the passage of ions and small molecules between cells, which is crucial for synchronized cellular activity (Orellana et al., 2013; González et al., 2007; Jindal et al., 2021). In humans, the connexin gene family includes 21 distinct members that encode the various connexins, each contributing to forming gap junctions that regulate cellular signaling and homeostasis (Beyer and Berthoud, 2018). Connexins can undergo modifications by factors such as pH, calcium levels (Ca^2+^), and phosphorylation, which influence the junctions’ conductance and selectivity (Jagielnicki et al., 2024). Such changes, especially during cardiac ischemia, may lead to gap junction closure, contributing to arrhythmias. In neurons, the flexible regulation of gap junctions impacts synaptic plasticity, playing a role in the development and adaptation of neural circuits (Rodríguez-Sinovas et al., 2021). Thus, gap junctions provide direct electrical coupling and integrate various physiological signals, finely tuning the electrical behavior of excitable cells. Figure 2A illustrates how hemichannels, made from connexins in cell 1 and cell 2, align to form a gap junction, enabling communication between the 2 cells. The red bidirectional arrow represents the cells’ ability to both send and receive signals at the same time. Figure 2B illustrates a linear arrangement of 6 cells linked by gap junctions, indicated by a red arrow, highlighting the directional flow of signals along this one-dimensional pathway. The lower section provides an electrical schematic representing the gap junction connection between 2 cells, labeled Cell 1 and Cell 2. Here, V1 and V2 indicate the membrane potentials of each respective cell, and r_j_ symbolizes the resistance within the gap junction between them.

In cancer, including BC, altered connexin expression and function can disrupt this communication. Loss of gap junction communication plays a key role in cancer progression and metastasis by disrupting cellular signaling that typically restrains abnormal cell growth. Furthermore, the activation of hemichannels—often separate from gap junctions—can release oncogenic molecules into the tumor microenvironment, thereby fostering conditions that promote cancer cell survival and invasiveness (Ouyang et al., 2024; Dominiak et al., 2020). The breast expresses several connexin family proteins, including Cx43, Cx30, Cx32, Cx46, and Cx26. Cx43, in particular, plays a role in the proliferation of mammary epithelial cells and the development of stromal tissue (Unal et al., 2022; Lee et al., 1991). Cx26 plays a significant role in the progression of ductal carcinoma *in situ* to invasive ductal carcinoma. It regulates cancer stem cell activity, which is crucial for tumor initiation, growth, and metastasis. (Zhang et al., 2024). Research indicates that mutations in Cx26 can cause deafness by altering the cell’s resting membrane potential (Stong et al., 2006). Research has shown that the absence of Cx43 gap junctions is a significant and independent marker for the presence of breast tumors (Laird et al., 1999; Teleki et al., 2014). The activation of Cx43 has been shown to rapidly depolarize the resting membrane potential and lower the input resistance of cells. These changes in cellular electrical properties are thought to play a role in the initiation of cancer by altering the normal signaling and behavior of the cell, potentially promoting tumorigenesis (Fasciani et al., 2013). Pharmacological strategies aimed at up-regulating Cx26 and Cx43 may help maintain the membrane potential at physiological levels. This stabilization could be beneficial in cancer treatment, as it may reduce the altered cellular electrical properties that contribute to tumorigenesis.

## 6 Interstitial cells of cajal and breast cancer

**FIGURE 3:**
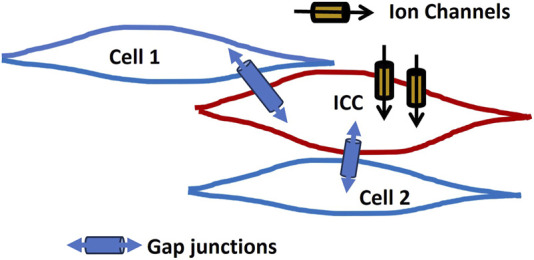
ICC network model. It presents a schematic of an ICC situated among smooth muscle cells. The ICC features multiple ion channels and establishes connections with neighboring cells via gap junctions. Detailed explanations are provided in the previous section.

Interstitial cells of Cajal (ICCs) are best known for their pacemaker function in the gastrointestinal tract, where they generate rhythmic electrical slow waves that regulate smooth muscle contractions (Sanders, 2019; Mostafa et al., 2010; Huizinga, 2018). ICCs express a variety of ion channels, including Ca^2+^, Na^+^, and K^+^ channels, which are essential for generating pacemaker activity to regulate the membrane potential. The important ion channels ANO1 (TMEM16A) and TRPM7 play crucial roles in the function of ICCs, contributing to their pacemaker activity and ion regulation (Dulin, 2020). These cells are connected by gap junctions, allowing them to form a coordinated network that propagates these slow waves through smooth muscle layers, ensuring proper motility (Daniel and Wang, 1999; Sanders et al., 2014). ICCs also play critical roles in other tissues, influencing smooth muscle activity in the urinary system, blood vessels, airways, and reproductive organs (Sanders et al., 2014). By modulating the membrane potential through ion channels and gap junctions, they help coordinate functions like peristalsis and vascular tone. Figure 3 presents a schematic representation of an ICC located between smooth muscle cells. The ICC is equipped with several ion channels and forms connections with neighboring cells via gap junctions.

It is now broadly recognized that ICC is likely the progenitor cells of gastrointestinal stromal tumors (Min and Leabu, 2006). These cells may contribute to changes in membrane potential that affect ion channels, potentially influencing processes like cell migration, proliferation, and metastasis. Interstitial Cajal-like cells have been suggested as potential components of the “permissive breast stroma,” a microenvironment that plays a role in cancer initiation and progression. However, there is still uncertainty regarding their definitive inclusion or exclusion as part of this stromal framework, indicating a need for further research to clarify their role in cancer biology (Gherghiceanu and Popescu, 2005; Cukierman, 2004; Huizinga et al., 1998).

## 7 Calcium dynamics and breast cancer

**FIGURE 4:**
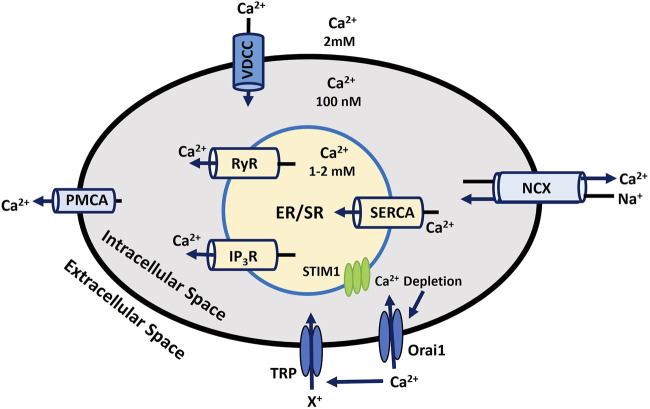
Intracellular Calcium Signaling and Homeostasis. This diagram depicts the regulation of calcium flux in the cell, emphasizing ER/SR Ca^2+^ storage, ion transporters, and key channels responsible for maintaining Ca^2+^ balance and cellular function. Detailed explanations are provided in the previous section.

Calcium dynamics and signaling are essential for various cellular functions, such as muscle contraction, neurotransmitter release, gene expression, and cell proliferation and differentiation regulation. Disruptions in Ca^2+^ signaling are linked to numerous health conditions, including cancer, neurodegenerative diseases, and cardiovascular disorders (Brini et al., 2013; Zündorf and Reiser, 2011; Stutzmann and Mattson, 2011). Ca^2+^ plays a vital role in maintaining the RMP by controlling ionic balance During membrane depolarization, Ca^2+^ influx is crucial for the depolarizing phase of the AP (Abdul Kadir et al., 2018; Mahapatra et al., 2024). Localized Ca^2+^ release events, such as Ca^2+^ puffs from inositol 1,4,5-trisphosphate (IP_3_) receptors and Ca^2+^ sparks from ryanodine receptors (RyRs) in muscle cells, are fundamental for initiating larger-scale cellular responses (Mei et al, 2014; Berridge, 2009). These localized events propagate across the cell, forming Ca^2+^ waves that help coordinate more extensive cellular activities (Guse et al., 2021; Demydenko et al., 2022). Intracellular Ca^2+^ levels are tightly regulated through the balance between Ca^2+^ influx via voltage-dependent Ca^2+^ channel (VDCC) and efflux through Ca^2+^-ATPase pumps (PMCA) and Na^+^- Ca^2+^ exchangers (NCX) across the plasma membrane (Firth and Yuan, 2011; Dayanithi et al., 2012). The sarco/endoplasmic reticulum (SR/ER) functions as a primary internal Ca^2+^ store in the cytosol (intracellular space). Ca^2+^ are released from these stores in response to signaling molecules such as IP_3_ and through RyR receptors (Bravo-Sagua et al., 2020; Eisner et al., 2013). This released Ca^2+^ activates downstream signals by binding to the targeting proteins, like Ca^2+^/calmodulin-dependent protein kinase (CAMK) and calcineurin, and eventually induces other targeted cellular processes (Anguita and Villalobo, 2018). The Ca^2+^-ATPase (SERCA) pump actively refills these stores by transporting Ca^2+^ back into the SR/ER using ATP, maintaining cellular Ca^2+^ homeostasis (Guerrero-Hernández et al., 2020). So, SERCA, PMCA, and NCX are crucial transport mechanisms that help keep Ca^2+^ levels low at rest. The complex interplay between these systems is also crucial for cellular responses to various internal and external stimuli, making Ca^2+^ signaling a central player in cellular homeostasis and function. When ER/SR Ca^2+^stores are depleted, it triggers the activation of Orai, STIM, and TRP ion channels (Roberts-Thomson et al., 2010). These channels then open in the plasma membrane, allowing Ca^2+^ influx from the extracellular space, a process critical for various cellular functions. This mechanism has been elaborated in earlier discussions in the ion channel section. Figure 4 depicts the processes involved in Ca^2+^ dynamics across all cellular and sub-cellular compartments, as outlined previously. The outward and inward arrow shows the flow of Ca^2+^ to/from intracellular space and ER/SR stores. The numerical Ca^2+^ concentration values in various compartments are taken from experimental findings (Görlach et al., 2015).

Calcium plays a vital role in breast milk, with its regulation being part of a carefully timed program (Olausson et al., 2012). During pregnancy, the expression and activity of Ca^2+^ transporters and modulators are adjusted (Mukherjee et al, 2023). These changes intensify during parturition when lactation begins, and they gradually diminish during involution, the final phase of the lactation cycle. This regulation ensures optimal Ca^2+^ levels are maintained for the infant’s growth and development (Zaragozá et al., 2015). In BC, the regulation of Ca^2+^ dynamics is disrupted, significantly impacting both membrane potential and intracellular Ca^2+^ concentrations (O’Grady and Morgan, 2021). Abnormalities in VDCC function, including altered expression or activity of VDCC channels, pumps, and binding proteins, lead to altered Ca^2+^ signaling pathways. These changes often result in elevated intracellular Ca^2+^ levels, which can affect cellular processes such as growth, proliferation, and apoptosis (Yang, Z. et al., 2020; Patergnani et al., 2020). Dysregulated Ca^2+^ influx and efflux contribute to changes in the RMP, facilitating tumor progression. Additionally, abnormal Ca^2+^ signaling is linked to tumorigenesis by promoting cell survival, metastasis, and resistance to cell death signals (Wang et al., 2013). Elevated Ca^2+^ levels can also cause altered gene expression and support the malignant transformation of cells, making Ca^2+^ dynamics a crucial factor in cancer development and progression (Makena and Rao, 2020).

## 8 Mitochondria and breast cancer

**FIGURE 5:**
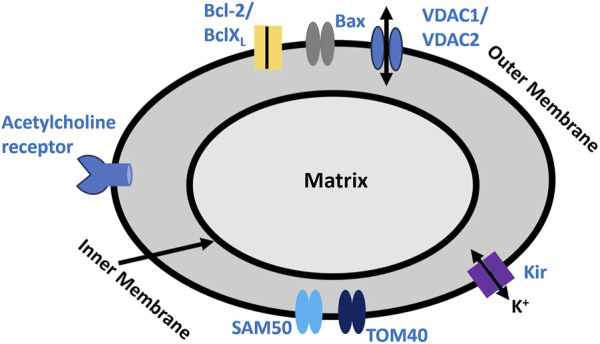
Mitochondrial Membrane Biophysics. It depicts proteins involved in mediating ion fluxes across the outer mitochondrial membrane, as described earlier. This schematic illustrates key ion channels and apoptotic regulators in the mitochondrial membrane, including VDACs, Kir channels, and protein transport complexes. It highlights the balance between pro- and anti-apoptotic factors, crucial for mitochondrial function and cellular homeostasis. It also includes the proposed acetylcholine receptor channel. Detailed explanations are provided in the previous section.

Mitochondria are crucial organelles in cells, primarily known for their role in ATP production via oxidative phosphorylation, a process essential for cellular energy, metabolism, and regulating programmed cell death (apoptosis) (Ott et al., 2007; Vakifahmetoglu-Norberg et al., 2017). Mitochondria play a key role in maintaining cellular redox balance and serve as primary sources of reactive oxygen species (ROS). Under certain conditions, mitochondrial dysfunction can elevate ROS levels, which contribute to breast cancer proliferation by inducing genetic mutations, activating oncogenic pathways, and altering the tumor microenvironment. In triple-negative breast cancer, creatine accumulation via the SLC6A8 transporter helps sustain redox homeostasis under hypoxia, reducing mitochondrial activity and ROS production while activating AKT-ERK signaling to enhance cell survival (Li et al., 2021). Additionally, the overexpression of peroxiredoxin 3 (PRDX3) regulates mitochondrial ROS, protecting cancer cells from oxidative stress (Whitaker et al., 2013). The thioredoxin-2 (TXN2) system also plays a crucial role in redox regulation and resistance to apoptosis, further supporting cancer progression (Seibold et al., 2011). The electron transport chain in mitochondria is made up of several protein complexes that transfer electrons, creating a proton gradient across the inner mitochondrial membrane (Billingham et al., 2022). The electrochemical gradient, referred to as the proton motive force, drives ATP production via ATP synthase (Cogliati et al., 2021). The mitochondrial membrane potential (ΔΨm) typically ranges from −150 to −200 mV, which is considerably more negative than the potential across the plasma membrane (Zorova et al., 2018). Mitochondria house several ion channels, such as the Mitochondrial Ca^2+^ Uniporter, Voltage-Dependent Anion Channel, Mitochondrial Permeability Transition Pore, and Mitochondrial K^+^ Channels (Peixoto et al., 2010). These channels play a crucial role in ATP synthesis by preserving the membrane potential and regulating ion and Ca^2+^ homeostasis. The ATP generated by mitochondria fuels ion pumps, such as the Na^+^- K^+^ ATPase, which maintains ion gradients and helps reset the RMP and AP. Furthermore, the release of Ca^2+^ from mitochondria influences ion channels, impacting membrane potential, Ca^2+^ dynamics, and cellular excitability (Rasola and Bernardi, 2011). Mitochondria are also a major source of reactive oxygen species (ROS), by-products of oxidative phosphorylation. While low levels of ROS function as signaling molecules, excessive ROS can damage cellular structures, including ion channels (Redza-Dutordoir and Averill-Bates et al., 2016). Figure 5 highlights the crucial ion channel components on the outer mitochondrial membrane. SAM50 and TOM40 are vital for protein import into the mitochondria, with TOM40 forming a channel that facilitates ion flow across the outer membrane (Dukanovic and Rapaport, 2011). SAM50 supports the assembly of this complex, ensuring the proper functioning of the channel. Acetylcholine receptors on the outer mitochondrial membrane create ion channels that regulate mitochondrial ion flow, impacting cellular function (Skok et al., 2016). Bcl-2 and BclXL proteins prevent apoptosis by modulating ion flow and blocking the release of pro-apoptotic factors, while Bax facilitates apoptosis by forming membrane pores (Qian et al., 2022). VDAC1 and VDAC2 also form ion and metabolite channels at the outer mitochondrial membrane, which are essential for energy production and signaling (Camara et al., 2017). K_IR_ channels control K^+^ flow, playing a crucial role in maintaining the mitochondrial membrane potential. Oxidative stress, especially from H2O2, can disrupt these proteins’ functions, impairing protein import, membrane assembly, and overall mitochondrial integrity (Brini et al., 2018).

Cancer cells exhibit an elevated ΔΨm, which is linked to their increased invasiveness in laboratory settings and a higher propensity for metastasis in living organisms (Begum and Shen, 2023). In one study. β-Sitosterol induces G1 arrest and causes depolarization of mitochondrial membrane potential and an increase in Bax/Bcl-2 ratio in breast carcinoma MDA-MB-231 cells (Vundru et al., 2013). Depletion of SAM50 at the mitochondria outer membrane targets BCR-ABL-Expressing breast Leukemic Stem and Progenitor Cells (Capala et al., 2016). TOM40 positively correlated with mitochondrial activities, and its association enhances the proliferation of ovarian cancer and BC (Zhou et al., 2024; Yang, W. H. et al., 2020). Mitochondria play a critical role in BC by influencing cell growth, invasion, and chemoresistance. Their metabolic activity, including mitochondrial dynamics and respiration, contributes significantly to tumor progression and metastasis.

## 9 The electrophysiological model of breast cancer cell

**FIGURE 6:**
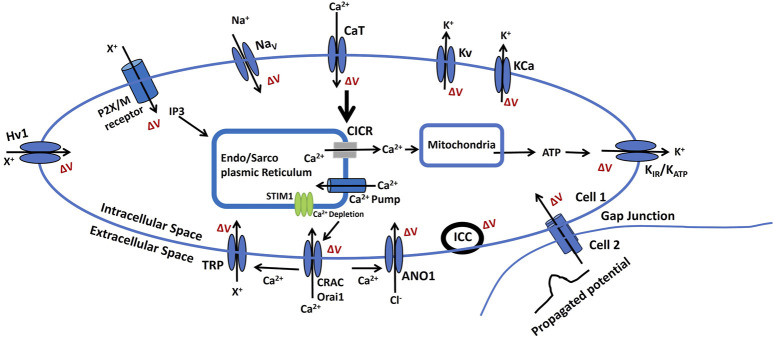
A schematic diagram of the BC cell electrophysiological model, It illustrates how ion channels and signaling pathways regulate small membrane potential changes (ΔV), resting membrane potential (RMP), and cellular excitability. Key components, including Na^+^, K^+^, Ca^2+^, and Cl^−^ channels, modulate CICR, ATP production, and intercellular communication via gap junctions. The role of the endoplasmic/sarcoplasmic reticulum (ER/SR), ion pumps, and receptors in maintaining bioelectric homeostasis is also highlighted. Detailed explanations are provided in the previous section.

Figure 6 depicts the changes in membrane potential through various pathways that influence cellular excitability. This electrophysiological model is grounded in the experimental analysis of the cellular and sub-cellular components discussed earlier. The ΔV is known as a change in membrane potential.

• Endo/Sarcoplasmic Ca^2+^ is stored in the endoplasmic or sarcoplasmic reticulum (ER/SR), an intracellular reservoir. Ca^2+^ ions are released into the sarcoplasm through Ca^2+^ channels regulated by intracellular signals. ATP-driven pumps restore Ca^2+^ levels in the ER/SR. A localized increase in Ca^2+^ concentration near the ER/SR triggers calcium-induced calcium release (CICR). Reactive oxygen species (ROS) can influence the mechanisms controlling Ca^2+^ storage and release in the ER/SR. Furthermore, Ca^2+^ regulates ATP and ROS production in mitochondria, while ROS can disrupt ER/SR Ca^2+^ dynamics. Released ATP may activate ATP-sensitive potassium channels (K_ATP_), including inward rectifier potassium channels (K_IR_), altering membrane potential.
• An increase in the concentration of a diffusible second messenger can connect surface membrane signaling to the release of intracellular Ca^2+^. This mechanism involves the activation of purinergic receptors (P2X) or M3 muscarinic receptors. These receptors trigger membrane-bound processes that generate inositol trisphosphate (IP3) upon activation. IP3 subsequently regulates Ca^2+^ dynamics as previously outlined. Alterations in the sensitivity or efficiency of this pathway can profoundly affect intracellular Ca^2+^ release. ATP binding to purinergic receptors (P2X/M) may open non-specific cation channels, facilitating the influx of positive ions (X^+^) and increasing membrane potential.
• Membrane potential can propagate from cell 2 to cell 1 via gap junctions, as certain excitable cells operate within a multicellular network or syncytium. Additionally, the activation of pacemaker interstitial cells of Cajal (ICC) can lead to an increase in membrane potential, contributing to coordinated cellular activity.
• The BK and IK/SK channels, classified as voltage-gated and Ca^2+^-activated K^+^ channels (KCa), enable the efflux of K^+^ from the intracellular to the extracellular space. This process results in hyperpolarization, altering the membrane potential. Similarly, voltage-gated K^+^ channels (Kv) allow K^+^ efflux, contributing to membrane repolarization. On the other hand, T-type Ca^2+^ channels (CaT), voltage-gated Na^+^ channels (Nav), and proton ion channels (Hv1) facilitate the influx of Ca^2+^, Na^+^, and nonspecific positive ions (X^+^), leading to membrane depolarization.
• Calcium release-activated channels (CRAC), part of the store-operated calcium channels (SOCs) family, including Orai1, are activated by intracellular calcium store depletion through STIM1 and STIM2. This activation facilitates Ca^2+^ influx, contributing to membrane depolarization. Additionally, Ca^2+^, along with other stimuli, can activate various TRP ion channels (discussed in the ion channel section), allowing the entry of cations (X^+^) and further depolarizing the membrane to regulate cellular excitability. Furthermore, Ca^2+^ also activates calcium-dependent chloride channels (ANO1), enabling Cl^−^ flux and enhancing membrane depolarization.

## 10 Therapeutic and clinical implications

Considering the critical role of membrane potential in regulating biological functions, especially in the context of BC, targeting ion channels and bioelectric signaling pathways offers a promising avenue for therapeutic innovation. Multiple strategies are currently under investigation.

### 10.1 Ion channels and transporters: use as cancer biomarkers in BC

Ion channels and transporters (ICTs) play a significant role as biomarkers and therapeutic targets in BC, with their activity closely tied to tumor type, progression, and severity (Lastraioli et al., 2015). Modulating ion channel activity through agonists or antagonists has shown potential in preclinical research for restoring normal cellular function and inhibiting cancer growth (Leanza et al., 2016). For instance, inhibitors of Kv10.1 and hERG channels have been shown to reduce proliferation and induce apoptosis in BC cells (Peretti et al., 2019). Similarly, targeting potassium channels such as KCNMA1, KCNJ3, and KCNN4, which are linked to metastasis and tumor grade, presents a promising strategy (Ganser et al., 2021). Sodium channel inhibitors, like those targeting SCN5A, may help curb metastasis, while calcium channels (e.g., SPCA1 and PMCA2) inhibitors are implicated in the proliferation of basal-like and HER2-positive BC subtypes (Lastraioli et al., 2015). TRP channels such as TRPM7, TRPV6, and TRPM8 display specific expression patterns across BC subtypes. Proton channels like Hv1 are associated with aggressive tumor characteristics. Studies, including the identification of the IC30 gene signature, emphasize the role of ICTs in predicting tumor behavior and prognosis. Dysregulation mechanisms include gene amplification (e.g., KCNK9), methylation (e.g., CACNA2D3), and hormonal regulation (e.g., ERα influencing ORAI3 and TRPM8). These insights highlight ICTs’ relevance in advancing BC diagnostics and treatment (Lastraioli et al., 2015).

### 10.2 Modulation of intracellular membrane potential

Innovative bioelectric therapies, including the use of electric fields to influence intracellular membrane potential, are being investigated as promising treatment options for BC. These techniques focus on disrupting the bioelectric signals that contribute to cancer progression, providing a targeted and non-invasive therapeutic approach (Zalba and Timo, 2017). Additionally, strategies to directly modify membrane potential are under development. This involves the application of drugs designed to either hyperpolarize or depolarize cancer cells, altering their behavior. These methods have the potential to enhance the effectiveness of current treatments and help address issues like therapy resistance.

### 10.3 Targeting mitochondrial membrane potential in cancer therapy

Research into developing drugs that specifically target cancer cell mitochondria is rapidly advancing. These therapies focus on leveraging the distinct metabolic and functional properties of mitochondria in cancer cells to either trigger apoptosis or suppress tumor progression. Agents known as mitochondrial uncouplers, which disrupt the mitochondrial membrane potential (ΔΨm), can impair ATP synthesis and activate apoptosis by releasing pro-apoptotic factors (Baffy, 2017). Targeting proteins that regulate mitochondrial dynamics, such as DRP1 (involved in fission) and MFN1/MFN2 (involved in fusion), can destabilize mitochondrial homeostasis in cancer cells, thereby reducing tumor growth and increasing the effectiveness of chemotherapy (Chen et al., 2023). Additionally, since cancer cells often depend on ROS signaling for proliferation, therapeutic approaches that alter ROS levels through mitochondrial targeting may induce oxidative stress and inhibit cancer cell survival.

## 11 Future directions and challenges

While the role of membrane potential in BC is an exciting and rapidly evolving field, several challenges remain. One of the primary challenges is the need for a deeper understanding of the complex interplay between membrane potential and cancer cell signaling pathways. Accurately measuring changes in cellular membrane potential is crucial for understanding various physiological processes.

Several techniques are employed for this purpose, each with specific detection limits and challenges.

• Electrode-Based Techniques: Methods like whole-cell patch clamp, cell-attached, and perforated patch configurations are considered the gold standard for recording membrane potential. They offer excellent temporal resolution and can quantify absolute membrane potential. However, these techniques are invasive, may become unstable over time, have low throughput, and provide limited spatial resolution (Lazzari-Dean et al., 2021).
• Optical Methods: Voltage-sensitive dyes enable the visualization of membrane potential changes. Nevertheless, factors such as variations in dye environment, loading efficiency, illumination intensity, fluorophore bleaching, and cellular morphology complicate fluorescence intensity measurements. These issues make calibration and determination of absolute membrane potential challenging, restricting optical analysis to detecting acute V_mem changes without reliable comparisons between cells or over extended periods (Lazzari-Dean et al., 2019).
• Magnetic Resonance Imaging (MRI): Recent studies have explored the feasibility of using MRI to detect membrane potential changes by measuring magnetic resonance parameters. Findings suggest that depolarization or hyperpolarization of the membrane potential can influence T_2 relaxation times and the ratio of bound to free water protons. While promising, this approach is still under investigation and not yet widely adopted (Min et al., 2024).

Additionally, the development of targeted therapies that can selectively modulate membrane potential in cancer cells without affecting normal cells is a significant hurdle. Future research should focus on elucidating the precise mechanisms by which membrane potential influences BC, identifying novel ion channels and bioelectric targets, and translating these findings into clinically effective therapies. Advances in bioelectric imaging and computational modeling may also provide new insights into the role of membrane potential in cancer biology.

• Advanced Imaging Techniques: Development of more sensitive and specific fluorescent probes and imaging techniques to measure real-time changes in membrane potential and ion channel activity in live cells and tissues (Khadria, 2022). This will enhance our understanding of the spatial and temporal dynamics of membrane potential propagation in the tissue.
• Integrative Multi-Omics Approaches: Combining genomics, proteomics, and metabolomics with electrophysiological data to construct comprehensive models of how membrane potential is modulated by ion channel function and impacts cellular excitability (Rudy et al., 2008). This holistic view can uncover new regulatory mechanisms and potential drug targets.
• Personalized Medicine: Investigating individual variability in a variety of agonist and antagonist responses and ion channel functions to develop personalized therapeutic strategies (Stevens and Stephens, 2024). Genetic and epigenetic factors that influence susceptibility to protein structures and ion channel modifications should be identified.
• Animal Models and Clinical Trials: Utilizing animal models to study the *in vivo* relevance of findings from cellular and molecular studies (Ioannidis et al., 2018). Translating these findings into clinical trials to evaluate the efficacy of targeted therapies in mitigating the effects of membrane potential in human diseases.
• Digital Twin and Computational Electrophysiology: Digital twin technology boosts breast cancer research by developing customized virtual representations of patients, allowing researchers to track disease progression and predict treatment responses (Awujoola et al., 2024). These virtual models enable the simulation of various therapeutic approaches, optimizing individualized treatment plans. Furthermore, digital twins facilitate predictive modeling to forecast treatment outcomes and identify the most effective strategies. By replicating real-world conditions, they enhance our understanding of tumor dynamics and treatment effectiveness. The computational electrophysiology approach uses mathematical simulations to model the electrical properties of cells and tissues (Dössel et al., 2012). By factoring in data like changes in ion channel conductance, these models can predict how alterations in membrane potential affect cell behavior and excitability.

## 12 Conclusion

Membrane potential is a critical regulator of BC progression, modulating key cellular processes such as proliferation, migration, and apoptosis. Dysregulation of ion channels and bioelectric signaling pathways contributes to oncogenic transformation, tumor aggressiveness, and therapy resistance, making these pathways promising targets for novel therapeutic interventions. This review synthesizes recent advancements in bioelectric membrane potential research, providing an integrated perspective on its role in BC pathogenesis. Unlike previous studies that examined ion channel function and cancer biology separately, this work explores their interconnection, shedding light on how bioelectric signaling governs metastasis, treatment resistance, and tumor evolution. It discusses innovative therapeutic strategies, including targeting membrane biophysics through ion channel modulators and bioelectric reprogramming, which could revolutionize precision oncology. Particular attention is given to aggressive BC subtypes, such as triple-negative breast cancer, where dysregulated membrane potential plays a pivotal role. Key molecular mechanisms, including ion flux alterations, mitochondrial dysfunction, calcium signaling disturbances, and impaired intercellular communication, are examined for their contributions to BC progression. Ion channels emerge as crucial therapeutic targets, with a deeper understanding of their role offering potential for restoring homeostatic bioelectric signaling. This review also explores cutting-edge approaches, such as computational modeling and digital twin technology, to advance personalized treatment strategies. By integrating experimental findings with predictive modeling, it highlights translational opportunities that bridge fundamental membrane biophysics with clinical oncology. Despite significant progress, knowledge gaps remain regarding the mechanistic underpinnings of membrane potential alterations in BC cells, necessitating further interdisciplinary research. Ultimately, this work underscores the importance of bioelectricity in cancer biology and lays the groundwork for developing targeted therapeutic strategies that address the root causes of membrane potential dysregulation in BC.
